# Autoencoder techniques for survival analysis on renal cell carcinoma

**DOI:** 10.1371/journal.pone.0321045

**Published:** 2025-05-15

**Authors:** Iñigo Sanz Ilundain, Laura Hernández-Lorenzo, Cristina Rodríguez-Antona, Jesús García-Donas, José L. Ayala

**Affiliations:** 1 Complutense University of Madrid, Madrid, Spain; 2 CSIC/UAM, Madrid, Spain; 3 HM CIOCC Madrid, Hospital Universitario HM Sanchinarro, HM Hospitales, Madrid, Spain; Hebei University of Technology, CHINA

## Abstract

Survival is the gold standard in oncology when determining the real impact of therapies in patients outcome. Thus, identifying molecular predictors of survival (like genetic alterations or transcriptomic patterns of gene expression) is one of the most relevant fields in current research. Statistical methods and metrics to analyze time-to-event data are crucial in understanding disease progression and the effectiveness of treatments. However, in the medical field, data is often high-dimensional, complicating the application of such methodologies. In this study, we addressed this challenge by compressing the high-dimensional transcriptomic data of patients treated with immunotherapy (avelumab + axitinib) and a TKI (sunitinib) into latent, meaningful features using autoencoders. We applied a semi-parametric statistical approach based on the COX Proportional Hazards model, coupled with Breslow’s estimator, to predict each patient’s Progression-Free Survival (PFS) and determine survival functions. Our analysis explored various penalty configurations and their combinations. Given the complexity of transcriptomic data, we extended our model to incorporate both tabular data and its graph variant, where edges represent protein-protein interactions between genes, offering a more insightful approach. Recognizing the interpretability challenges inherent in neural networks, particularly autoencoders, we analyzed the mutual information between genes in the original data and their latent feature representations to clarify which genes are most associated with specific latent variables. The results indicate that different types of autoencoders are better suited for different tasks: denoising autoencoders excel at accurate reconstruction, while the sparse variant is more effective at producing meaningful representations. Additionally, combining these penalties enhances both reconstruction quality and the interpretability of latent features. The interpretable models identified genes such as LRP2 and ACE2 as highly relevant to renal cell carcinoma. This research underscores the utility of autoencoders in managing high-dimensional data problems.

## Introduction

Kidney cancer is the 12th most common type of cancer worldwide, accounting for 2.4% of all cancers, with over 330,000 new cases every year. The most frequent type of kidney cancer is renal cell carcinoma (RCC), representing over 90% of all cases [1, 2]. The early diagnosis of RCC is crucial for effective treatment and reducing mortality rates [3]. However, the risk factors underlying RCC are still unclear due to the complexity and heterogeneity of disease [4].

Although there are difficulties surrounding early diagnosis and characterization, several treatments for RCC exist, such as immunotherapies, which can be targeted towards certain cells of the immune system, or chemotherapies, which target all rapidly dividing cells. However, considering the complexity and heterogeneity of RCC, detecting which treatment or combination of treatments is most appropriate for each patient’s case is an important challenge within personalized medicine that has yet to be solved.

The concept of personalized medicine is based on the idea that not only tumor genetic and molecular characteristics but also patient physiological, and environmental conditions should be considered when tailoring the interventions they should receive [5]. In this regard, data from omics technologies play an essential role in case characterization. Among these technologies are transcriptomics data, which consist of positive continuous variables that indicate the frequency of gene expression in their cells. Gene expression profiling is a powerful method to measure gene activity, especially during the onset of cancer [6]. In this sense, gene expression data has been successfully used to predict and characterize the response to specific treatments in cancer [7].

Furthermore, when complex diseases, such as RCC, are studied, it is necessary to couple the omic data with the relationships between the different actors that play a role in the development of the disease. This is where network biology strategies come into play, to study more holistically the genetic and molecular architecture of a disease [8, 9]. Among the potential biological interactions, the most known and common ones are protein-protein interactions (PPI). These data represent the physical interactions between the proteins encoded by the genes considered. These interactions can be represented in a network where genes are no longer independent, and information flows through these interactions. Therefore, in this work, we propose combining transcriptomics and PPI data to characterize the response profile to specific RCC treatments.

To characterize this response profile, it is necessary to study the patients’ survival. Usually, an effective solution to predict a patient’s survival for a specific treatment is using statistical methods, such as regression models. These models can integrate a series of covariates and learn coefficients based on the importance of the features. However, these methods are often employed on low-dimensional data [10], which is not the case regarding gene expression data, which is high-dimensional. This problem is even more acute when compared with the sample size, which is usually low in clinical trials. Additionally, these datasets usually contain noise and missing values which can hinder the inference of the statistical models. To solve this, an efficient way to handle high-dimensional data is through dimensionality reduction techniques. In this regard, machine learning offers a variety of valuable methodologies from which we highlight autoencoders (AEs). Autoencoders are a type of deep neural network that compresses data into lower-dimensional numerical representations while retaining essential features, which can prove an exciting method for transcriptomic data. Compared to other dimensionality reduction techniques, AEs can represent non-linear relationships that could appear between features, which is especially important in the study of complex data such as transcriptomics. More precisely, it specializes in extracting robust features through the use of non-linear activation functions that other methods do not possess [11].

Furthermore, because we proposed to include interactions data, we introduce *graph autoencoders* (GAEs) [12], a type of AEs that can include graph-structured data to obtain new numerical representations of it.

Therefore, in this work, we propose an analysis strategy for predicting survival in several clinical trial cohorts, employing transcriptomic data, biological interactions data, and AEs for feature extraction. The main objective of this work was focused on detecting, if possible, the transcriptomic profile behind the positive or negative response to each treatment. In addition, we included as secondary objectives: (i) the comparative use of the latent variables obtained from different AEs architectures and employing or not information of biological interactions between the genes of interest, and (ii) the comparison between survival of the different RCC cohorts considered, treated with different treatments.

The structure of the presented work is as follows. The Materials and methods section will elaborate on the data and methodology utilized to unravel the proposed objectives. The Experimental results section will detail the results achieved by applying the methodology, and the Discussion section will discuss these findings. Finally, the Conclusion section will draw the main conclusions obtained from this work.

## Materials and methods

In this section, we will review the data we used, how we preprocessed it, the creation of the PPI network, and its integration with gene expression data. Finally, we will explain our design choices and the implementation of several autoencoder architectures and statistical models for predicting survival.

### Data preparation

#### Data description.

The data used in this study are derived from the JAVELIN Renal 101 trial [13], a randomized phase 3 trial. The primary aim of this trial was to investigate and compare the efficacy between two cohorts: an immunotherapy regimen involving avelumab and axitinib, and a tyrosine kinase inhibitor (TKI) named sunitinib. The trial included 886 eligible patients, all diagnosed with previously untreated renal cell carcinoma, over 18 years of age, and randomly assigned to the immunotherapy or sunitinib cohort. Of these 886 patients, 726 have expression data of 22,955 genes, represented by positive real values.

**Fig 1:**
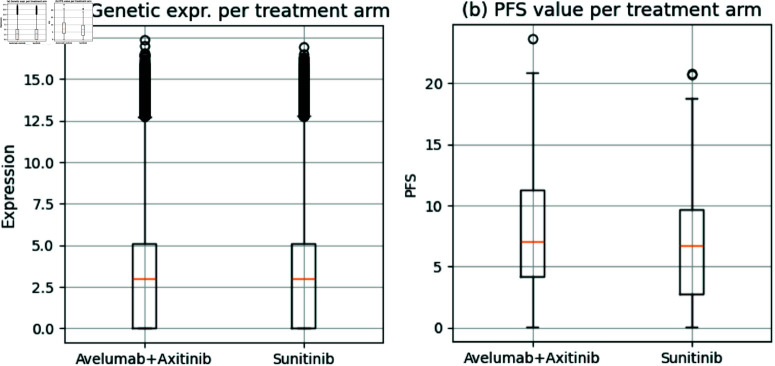
Boxplots showing the distribution of (a) gene expression and (b) PFS among the considered cohorts.

Additionally, six histology measurements were available for the patients. These measurements include metrics such as the percentage of cancer cells in the tumor area and the number of infected cells in the invasive margin. It is crucial to note that all these measurements are taken before treatment, ensuring the treatment itself does not influence them. Demographics (including the patient’s age and sex) and survival information are also recorded for each patient. Survival information included *Progression-Free Survival* (PFS), the amount of time for which the patient’s condition has not reached a particular event, such as death [14], and *censoring status*, which indicated if the event occurred [14]. [fig:gen+pfs_dist]Fig 1 shows the distribution of gene expression values and PFS in the two cohorts (avelumab+axitinib and sunitinib) considered.

Both cohorts were very similar in several aspects. Avelumab+axitibib cohort included 354 patients, of which 157 (44.35%) were censored. On the other hand, the sunitinib cohort included 372 patients, with 171 (45.96%) censored. Moreover, Fig [fig:gen+pfs_dist]1a displays that both cohorts had very similar gene expression distributions and should not introduce any bias in the model’s predictions from a data analysis standpoint. Specifically, Avelumab+axitinib cohort has a genetic expression average of 2.98 ± 2.63, whereas sunitinib has an average expression of 2.98 ± 2.64. Similarly, PFS values distributions were very similar ([fig:gen+pfs_dist]Fig 1b), further indicating that the two cohorts were comparable in survival.

#### Data filtering and preprocessing.

The JAVELIN Renal 101 trial dataset contains 22,955 genes. Many of these genes are not relevant to the development of the disease or are expressed only in a small subset of patients. Considering irrelevant diseases will only add noise to the treatment response of the model. We were able to gauge which diseases could be considered relevant or not using DisGeNET [15], as we will demonstrate shortly. This fact might introduce noise that can complicate the model’s inference based on the patient’s overall genetic expression. Several filtering steps were applied to reduce this noise, explained in the following lines.

1. **Gene filtering**. We removed genes specific to certain individuals, characterized by low expression in most patients and higher expression in a small subset. Let *G* be the set of genes expressed in each patient, and *X*_*g*_ be the expression for a gene g∈G. The genes removed satisfied the following condition:
  $$\forall g\in G:\left(\text{median}(X_{g})<2\&Q3(X_{g})<4\right)$$
  Thus, we excluded genes with a median expression lower than 2 and an upper quartile lower than 4. These values were chosen with the goal of achieving a graph with strong connectivity. Increasing them further would result in a very limited number of genes and lead to a sparse graph.
2. **Integration of histology features**. Since the histology features available were all pre-treatment, they were integrated to enhance the statistical model’s inference. Out of the seven histology features, only two were measured in all patients: “he_tumor_cell_content_in_tumor_area” and “PD-L1_total_immune_cells_per_tumor_area,” measuring the percentage of cancer cells and the number of PD-L1+ cells in the tumor area, respectively. Only these two features were included to avoid further reducing the sample size.
3. **Selection of RCC-Specific Genes**: To further refine the dataset, genes specific to RCC were selected using DisGeNET [15], a platform that aggregates information on human disease-associated genes and variants. Gene-disease associations were queried for RCC (CUI: C0007134) and three related conditions: conventional (clear cell) RCC (CUI: C0279702), hereditary RCC (CUI: C2608055), and familial RCC (CUI: C2931352). This resulted in a total of 4,447 gene-disease associations (GDAs), corresponding to 2,582 unique genes, represented in *symbol* format, a type representation for genes. When intersected with the original expression data, the total number of relevant genes was reduced to 2,403.

We also applied several pre-processing steps. Regarding the features, which consist of genetic and histology variables, we normalized the genetic expressions based on the maximal global expression value. This is because transcriptomics is based on counting, ensuring that variations in gene expression levels are accounted for. For the histology variables, traditional normalization was applied.

As for the labels, since the PFS value is measured in months, we converted it to trimesters to facilitate better clinical analysis.

As a result of preprocessing, we ended up with 726 patients, 354 in the avelumab+axitinib arm, and 372 in the sunitinib arm, where each patient has 2,403 genes.

### Protein-protein interaction network

Having obtained a relevant set of genes associated with the disease, we decided to build a PPI network, a graphical representation of the physical interactions between proteins within a biological system. This representation is intended to give context to the model on how information flows through the different genes in our dataset. We obtained the PPI data from PPT-Ohmnet, an interaction network from the Stanford Network Analysis Platform (SNAP) [16]. PPT-Ohmnet has a collection of physical PPI networks across different human tissues. In this network, proteins are represented as nodes, and the edges are the physical interactions between proteins.

Given the nature of RCC, we selected the sub-network that includes only kidney tissues. This subnetwork consists of 3,304 nodes and 52,126 edges. Due to the network being generic to the kidney, we still needed to perform feature selection to only obtain those genes relevant to RCC. As such, using the 2,403 genes obtained in Section [subsec:dataprep], all relevant to RCC, we retrieved the related kidney-specific interactions, removing self-loops and disconnected components smaller than five nodes. Genes in PPT-Ohmnet are represented in *Entrez ID* format, a different form of representation than the genes queried from DisGeNET. For this reason, we translated between different gene representations using a Python library called mygene [17].

**Fig 2:**
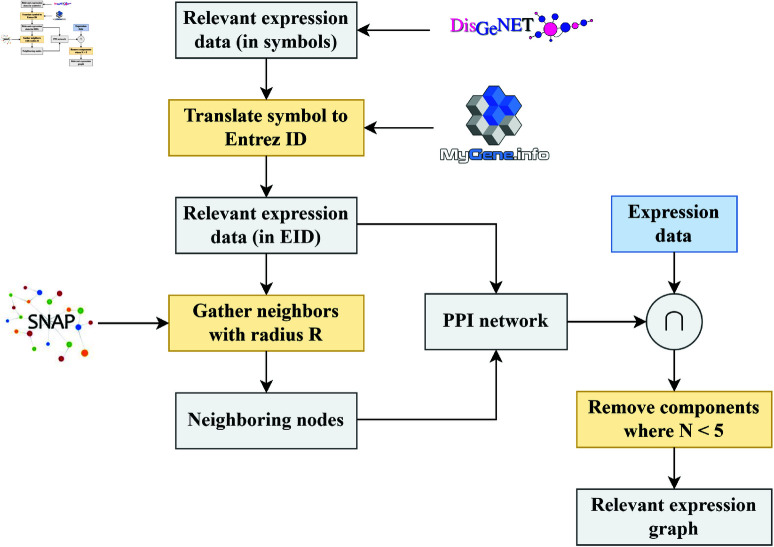
Visualization of the pipeline to create the PPI networks associated to each patient.

We retrieved 837 nodes. Given the limited number of nodes, we created a recursive neighbor lookup with a depth of 3 to add similar genes to the gene pool using breadth-first search. This resulted in a PPI network consisting of 2,865 nodes, where each node holds the expression level of a given gene. Fig 2 shows this pipeline in more detail.

We performed a graph analysis to ensure that no meaningful bridges existed within the graph. A bridge would indicate that the graph is comprised of two or more large components, affecting the flow of information. Additionally, we calculated the betweenness and closeness centralities of the graph, producing a mean and standard deviation of $5.90\times 10^{-4}\pm 0.02$ and 0.37 ± 0.04, respectively. A betweenness of 1 would suggest that a path in the network has a large influence on the flow of information, and a closeness of 1 would indicate that nodes are, on average, very close to each other. These values indicate that we have an interconnected graph with multiple paths where information can flow and where all nodes are not adjacent to eachother.

With the PPI network ready, we created one for each of the 726 different patients. The networks between patients are identical except for the node attributes, which hold different expression levels for each gene, depending on the patient. In addition, the graphs are attributed with the histology features aforementioned for each patient.

### Autoencoder embeddings

An autoencoder is a neural network designed to represent high-dimensional data in a lower-dimensional space. It operates under the assumption that high-dimensional data reside in or near a low-dimensional manifold within the input space [18]. Autoencoders consist of two components: an encoder *f*_*e*_ that compresses the data $𝐱\in {\mathbb{R}}^{d}$ into a low-dimensional representation $𝐳\in {\mathbb{R}}^{p}$ (where *d* > *p*), and a decoder *f*_*d*_ that performs a reconstruction $\hat{𝐱}\in {\mathbb{R}}^{d}$ from **z**. These components are trained to minimize a loss function that measures the quality of the reconstruction [19, p. 677]:


$$ℒ(f_{e},f_{d},𝐱)=(\hat{𝐱}-𝐱{)}^{2}=(f_{d}(f_{e}(𝐱))-x{)}^{2}$$

Standard autoencoders can be extended to regularized variants in order to achieve specific tasks such as a better generalization or more meaningful representations [20].

The variants we use in this work are:

- A denoising autoencoder [21], which introduces a controlled amount of noise ϵ~𝒩(0,1) to the input, yielding a noisy version $\tilde{𝐱}=𝐱+\epsilon$.
- A sparse autoencoder [22] aims to reduce as much as possible the magnitude of the weights used in the neural network. We chose KL divergence as our sparse variant, as it aligns the activation distribution with a target distribution [23]. Specifically, it measures the divergence between two Bernoulli distributions: one with the observed activation probability and the other with the desired activation probability.
- A variational autoencoder [24] regularizes the latent space, rendering it continuous and making the model generative. Instead of mapping input data to a latent space, the variational autoencoder models its distribution and samples from it via the reparameterization trick [24].

These variants can be combined to further enhance latent representations [25].

Additionally, autoencoders are applicable to graph-structured data. Graph Autoencoders (GAE) take a graph G=(V,E), with *V* as nodes and *E* as edges, and $A\in {\mathbb{R}}^{N\;\times \;N}$ as the adjacency matrix. The aforementioned regularization techniques are also viable in GAEs, targeting either the graph’s features, its adjacency matrix, or both [26].

### Statistical approaches

Utilizing survival analysis allows us to make inferences and predictions regarding the survival of new patients.

In this context, each patient holds a pair of values (*y*_*i*_, $\delta_{i}$) representing the patient’s PFS and censoring value, respectively.

Censoring in a patient, denoted by $\delta_{i}\in 0,1$ is a binary indicator reflecting whether the event has been observed. In the context of RCC, $\delta_{i}=1$ indicates that the i-th patient did not show progression of cancer during treatment.

Consequently, the PFS of a patient represented as $y_{i}\in 𝐑$, is a measurement in months of how long no cancer progression has been observed. It can be interpreted as:


$$y_{i}=\left\{ \begin{array}{cc} t & \text{if }\delta_{i}=1\\ c & \text{if }\delta_{i}=0 \end{array}\right.$$

where *t* is the *event time* when an event occurred, and *c* the *time of censoring*.

Patient survival is characterized by a survival function *S*(*t*, *X*), which models the probability that an event has not occurred by time *t*, and a hazard function *h*(*t*), the instantaneous likelihood of an event occurring at time *t* since no event has occurred before time *t*. The choice of method depends on the distribution of the data. Given the complex nature of RCC data and the lack of assumptions we can make about its distribution, semiparametric models, particularly the COX Proportional Hazards (PH) model, are suitable choices.

In the context of the COX PH model, the survival and hazard functions are defined as follows:


$$S(t,X)=S_{0}(t{)}^{\exp(X\cdot \beta )}\;h(t|X)=h_{0}(t{)}^{\exp(X\cdot \beta )}$$

where β is an array of coefficients associated with the covariates *X*, *S*_0_(*t*) is the baseline survival function, and *h*_0_(*t*) is the baseline hazard function.

A significant advantage of the COX PH model over other models is that to find the hazard ratio between patients, $\frac{h(t|p_{A})}{h(t|p_{B})}$, we can omit *h*_0_(*t*). This allows us to determine the risk between patients without making assumptions about *h*_0_(*t*).

Estimating the survival function *S*(*t*, *X*) for a patient involves estimating $S_{0}(t)=\text{exp}(-H_{0}(t))$, where *H*_0_(*t*) is the baseline cumulative hazard function. We can use Breslow’s nonparametric estimator to estimate *H*_0_(*t*) and thereby *S*(*t*, *X*) [27]. This estimator is commonly used in Cox PH models when no specific form for the baseline hazard function is assumed.

The COX PH model has a regularized variant that incorporates both Lasso ($\ell_{1}$ norm regularizer) and Ridge ($\ell_{2}$ norm regularizer) penalties, combined to form an Elastic Net, which is beneficial for feature selection [28].

The Elastic Net formulation is given by


$$\lambda \left[\alpha \sum_{p=1}^{P}|\beta_{p}|+\frac{1}{2}(1-\alpha )\sum_{p=1}^{P}\beta_{p}^{2}\right]$$

where λ is the regularization parameter controlling the overall strength of the regularization, α gauges the balance between the $\ell_{1}$ and $\ell_{2}$ regularizations, *P* is the total number of features, and β are the coefficients obtained in the COX PH model.

### Design

**Fig 3:**
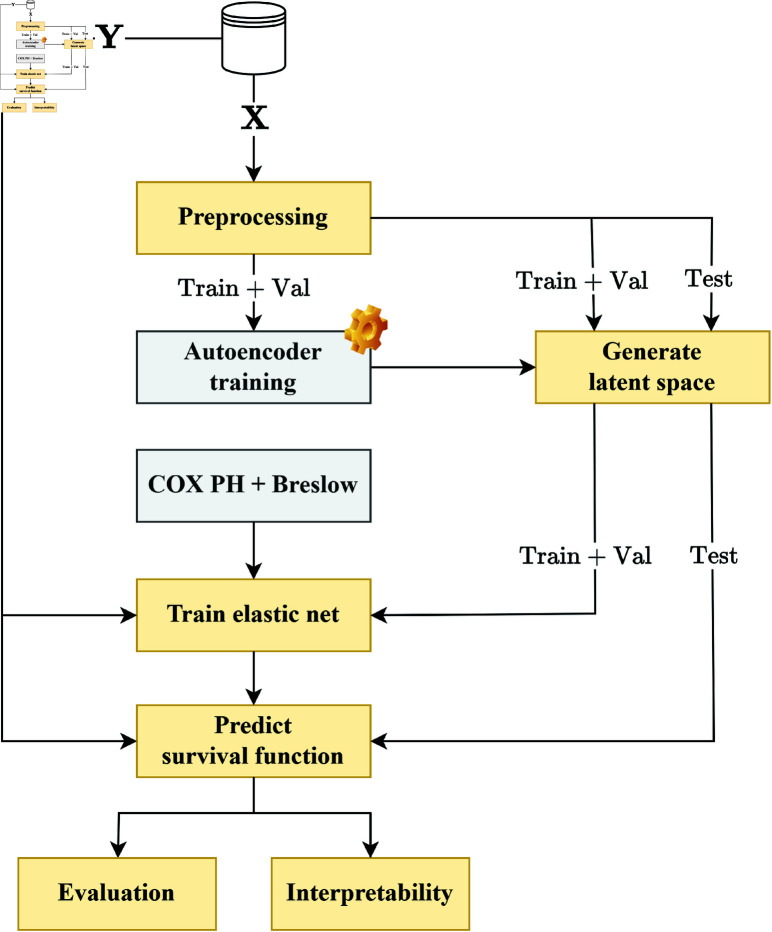
Pipeline for training and evaluating the models. **X** represents the genetic and histology data, while **Y** represents the set of PFS and censoring values for the patients.

The models developed for this work all follow the same pipeline, seen in Fig 3. They compress the data using an autoencoder, and once the representation is learned, the latent representation is fed to the COX PH model, which computes the hazard ratios of the patients and returns the coefficients assigned to each feature. This model is combined with Breslow’s estimator [27] to estimate survival functions for prediction.

The key difference between the models lies in the type of autoencoder used. Two different autoencoders were developed for this work: (i) a tabular autoencoder, which considers only tabular data where the genes are not placed within a context and can be freely combined, and (ii) a graph autoencoder, which considers the previously mentioned PPI network and reconstructs the features based on the context provided by the graph. Both autoencoders compare the Mean Squared Error (MSE) between the original and reconstructed features.

We incorporated cross-validation (CV) in our model generation process. During CV, we further divided the data of the training folds into 85% for training and 15% for validation. The autoencoders were trained using the training set, and to prevent overfitting, we evaluated the models on the validation set. Finally, the remaining fold was used for testing.

**Fig 4:**
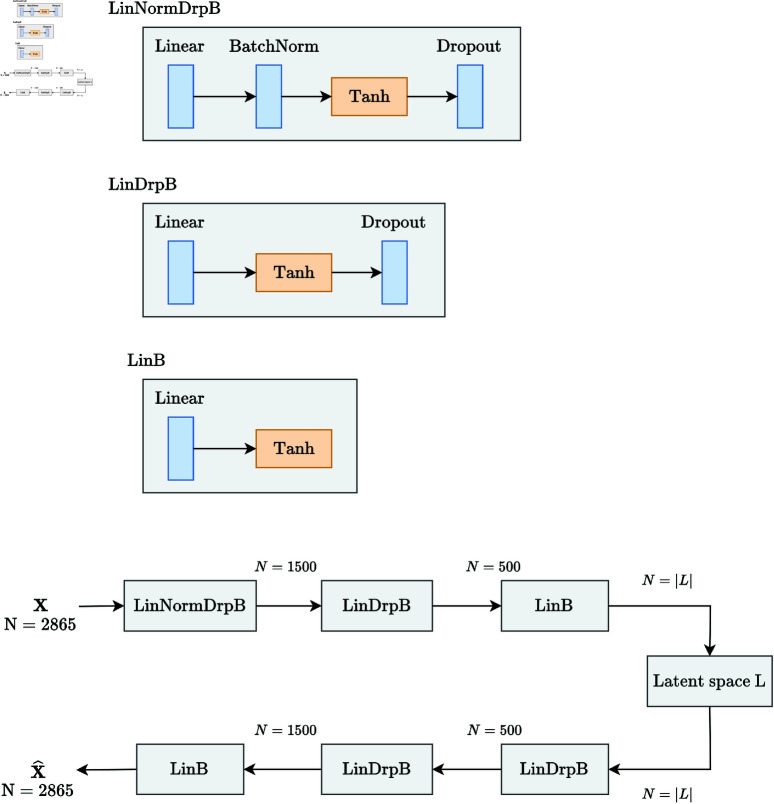
Architecture of the tabular autoencoder.

The architecture of the tabular autoencoder can be seen in Fig 4. All blocks used combine linear layers in order to reduce the dimensionality of the input, along activation functions such as tanh and sigmoid. The sigmoid activation function was employed in order to obtain a latent representation with values between 0 and 1, which could then be concatenated with the histology features, which are normalized as well.

Additionally, a BatchNorm layer [29] was added to normalize the data to a mean of 0 and unit variance. This step significantly improved the generalization process. Finally, dropout layers, where 20% of weights are set to zero, were added to prevent the model from overfitting to the training data.

**Fig 5:**
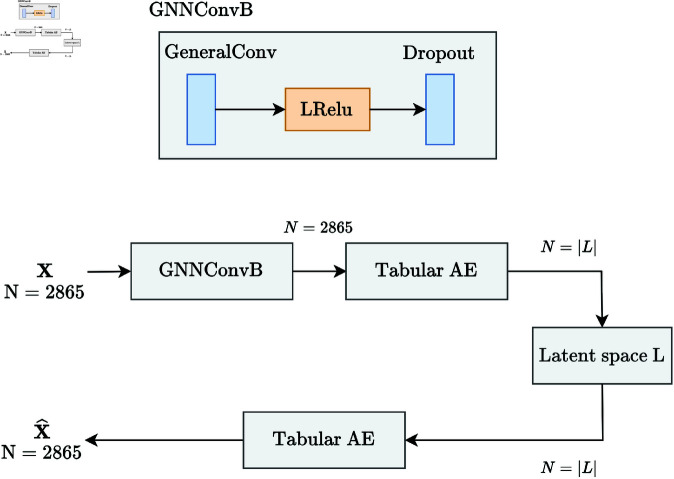
Architecture of the graph autoencoder.

The graph autoencoder is slightly more complex than the tabular one (Fig 5). The input graphs, obtained using the NetworkX [30] library, are processed through a GeneralConv layer consisting of several message passing layers, which reportedly yield better results than the default GCN layer [31].

Similarly to the tabular autoencoder, we add a dropout layer with a rate of 10%. Once the graphs are processed through the layer, the information flowed through the connections present in the PPI network. We then achieve a compression of the data by using the tabular autoencoder defined in Fig 4. As mentioned in the Autoencoder embeddings section, among the different reconstruction options, we chose to reconstruct the features, as we are particularly interested in obtaining a representation of the gene expression levels rather than the connections in the PPI network.

For autoencoder hyperparameter tuning, we performed a grid search to determine the optimal parameter combination for the model. Rather than exploring the entire range of possible values, we constrained the search space to avoid excessively high noise levels in the denoising autoencoder and overly weak regularization in the sparse autoencoder, as both could compromise the quality of the learned representations.

As for the statistical model, we train the COX PH model with the latent representation of both the training and validation sets concatenated, since its implementation does not include a validation process [32]. We explained in section [subsec:stadistical-methods] that the elastic net incorporated to the COX PH model uses a hyperparameter λ for regularization. To find the optimal λ, we performed a grid search in the set $\left[10^{-4};10^{-2}\right]$, with a step of $2\;\times \;10^{-4}$. We keep the λ for which the Concordance Index IPCW is maximal with the training and validation sets combined. To improve the regression of the statistical model, we standardize the data so that it has a mean of zero and unit variance. Once the COX model is trained, we use the test set to obtain the survival functions and the area under the ROC curve, utilizing the optimal λ value identified earlier.

### Implementation

Both autoencoders were implemented with a batch size of 16 samples, for a total of 100 epochs, with a learning rate of 1 × 10^−4^, which decays by a factor of 0.5 every 25 epochs, and latent dimensionalities of 16,64,128. Regarding the penalties of the autoencoders, the denoising autoencoder applies a noise input of 0.1% to the normalized data. Adding more noise deteriorates the quality of the encoding due to the low amount of samples. The sparse autoencoder uses a regularization strength of 10% and a parameter ρ of 1 × 10^−3^. To speed up the training process, GPU compatibility was added to the autoencoders.

For the COX model with elastic net, we used the implementation in the scikit-survival 0.22.2 [32] Python library, selecting a regularization ratio of α=0.5 to balance the $\ell_{1}$ and $\ell_{2}$ regularizations. We initially experimented with different weight distributions for the regularization terms. However, increasing the $\ell_{1}$ penalty led to the removal of too many features, while a higher $\ell_{2}$ penalty resulted in excessively small coefficients. To balance these effects, we opted for an intermediate approach. A grid search was performed to find the best regularization term λ, within the interval $\left[10^{-4};10^{-2}\right]$, with a step of $2\times 10^{-4}$. The search focused on the best concordance index IPCW, a modification of the original concordance index that provides a better estimate when censored data is present [33]. Additionally, a stratified K-Fold with *K* = 7 was used to ensure an equal number of observed samples in both the train and test sets. Once the COX model returned the coefficients associated with each covariate, Breslow’s estimator was used to obtain the survival functions. The PFS prediction was obtained using the area under the survival function.

Finally, we interpreted the results obtained by the model. To achieve this, we utilized mutual information, a metric that quantifies the dependency between random variables. A value of 0 indicates complete independence, meaning the variables share no information, while a value of 1 signifies total dependence, where knowing one variable entirely eliminates uncertainty about the other. [34] We evaluated the mutual information between the five latent features with the highest weights, as determined by the semi-parametric model, and the original transcriptomic data. This evaluation allowed us to identify the genes most represented within the latent representations, which were subsequently chosen by the statistical model.

## Experimental results

### Experiments

We studied how different autoencoder types and penalties performed in predicting PFS and risk scores of new patients. For both tabular and graph autoencoders, we evaluated the performance of the sparse, denoising, and variational penalties, as well as the combination of sparse and denoising penalties.

For each model, we obtained a series of metrics:

- **Autoencoder Reconstruction**: Measures how well the autoencoder can reconstruct the input data.
- **MSE between Actual and Predicted PFS**: Evaluates the accuracy of PFS predictions.
- **Mean of the Area Under ROC**: Indicates the overall quality of risk assignment by the model.
- **Overestimation of PFS Predictions**: The percentage of times the predicted PFS is higher than the actual PFS of the patient, where 0% indicates no overestimation.

The first two metrics are values in the range [0;∞), where a value of 0 represents a perfect match in either the reconstruction or the predictions. The mean of the Area Under ROC is a value in the range [0;1], with a higher value representing better risk assignment by the model. The overestimation metric indicates how often the model overestimates the patient’s survival, and thus a percentage of 0% would indicate that the model never overestimates the patient’s survival.

Since we are working with autoencoders, we experimented on different latent dimensionalities, specifically 16, 64, 128.

To ensure the validity of the results, we performed a 10-fold cross-validation. We also conducted an analysis of variance (ANOVA) between the results obtained by the tabular and graph autoencoders and the different autoencoder penalties used, assuming a significance level of 0.05. Additionally, we ran the analysis on the different treatment arms to determine whether the model performed better for avelumab+axitinib or sunitinib. Finally, we compared our methodology to a PCA model to compare the efficiency of a linear versus a non-linear model in this scenario. We also experimented with using a distribution other than Gaussian for the variational autoencoder, specifically an exponential distribution.

### Results

We obtained results for different latent dimensionalities, specifically L={16,64,128}, to determine how much we could compress the data without losing valuable information. Out of these three dimensionalities, we chose to elaborate on 64 latent features, not only because of the significant compression of the original data but also because the results were very similar to those with higher dimensionalities.

**Fig 6:**
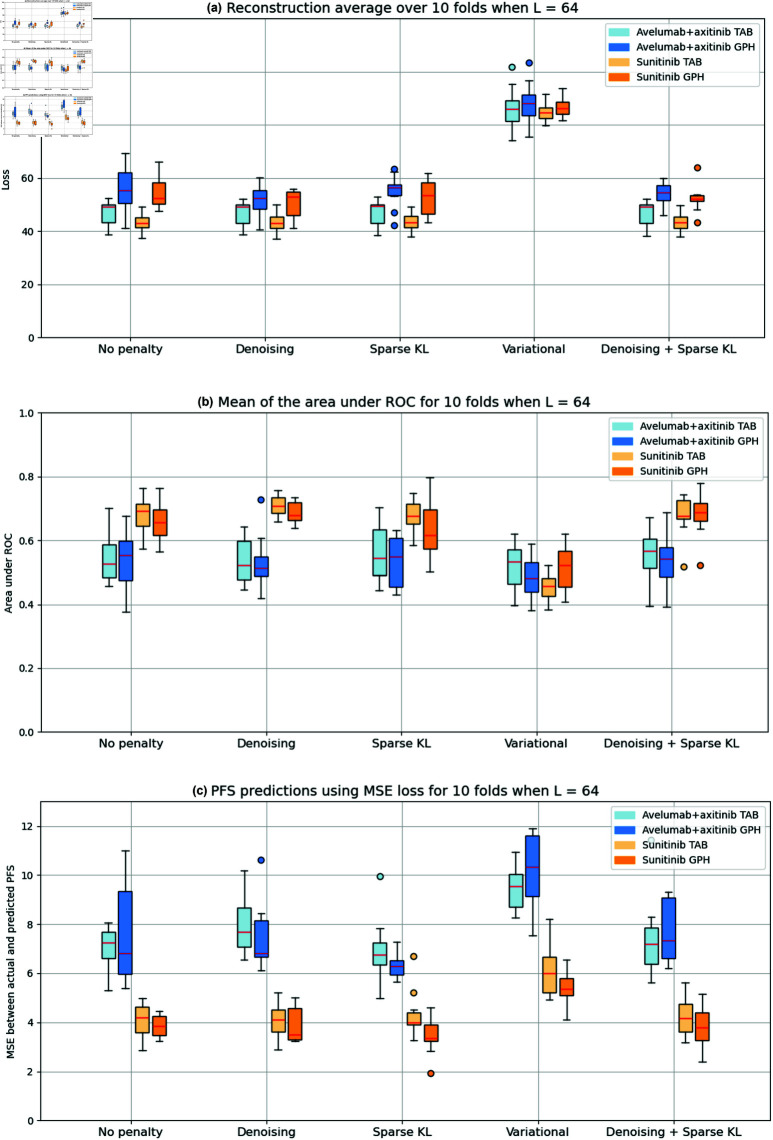
Results for (a) reconstruction, (b) mean of the area under ROC, and (c) PFS prediction for each type of autoencoder over 10 folds when *L* = 64.

In Fig 6, we can see the overall results obtained for *L* = 64 over 10 folds. We gathered the best results obtained for each autoencoder type and treatment arm in Table ??.

Firstly, Fig 6a shows the reconstruction loss of the autoencoder relative to the original transcriptomic data. Regarding the tabular autoencoders, the reconstructions over the different folds are very similar, with the best performance by the denoising autoencoder, having a mean and standard deviation of 43.52 ± 3.51 in the sunitinib arm. Meanwhile, the variational autoencoder performed the worst, with a loss of 86.05±8.00 in the avelumab+axitinib arm. The reconstruction performed by the variational autoencoder was significantly different from the autoencoder with no penalty for both the avelumab+axitinib (*p* = 1.88*e*^−10^) and the sunitinib arm (*p* = 1.7*e*^−15^).

The graph autoencoders did not reconstruct the data as well as their tabular counterparts, with the best reconstruction obtained by the denoising variation, achieving a loss of 50.43 ± 5.30 in the sunitinib arm, and the worst reconstruction by the variational autoencoder, with a loss of 87.97 ± 8.05 in the combination arm. As previously noted, the variational autoencoder was significantly different in both the immunotherapy (*p* = 1.8*e*^−7^) and the TKI (*p* = 8.7*e*^−11^) arms. There was a significant difference between the tabular autoencoder with no penalty and the graph autoencoder with no penalty for both the avelumab+axitinib (*p* = 1.1*e*^−2^) and sunitinib (*p* = 1.0*e*^−4^) treatment arms, indicating that the tabular autoencoder is indeed better at reconstructing the original data. No differences were observed between treatment arms. Sampling from an exponential distribution, as opposed to a Gaussian distribution, resulted in a 25% degradation in reconstruction performance when compared to a Variational Autoencoder with a Gaussian prior. The autoencoder reconstruction results using an exponential distribution are provided in the Appendix S1 Fig.

Regarding Fig 6b, we see the mean of the time-dependent area under ROC for each type of autoencoder. As defined in Section [sec:mats_methods], this metric is an overall estimate of how well the statistical model assigns risks to each sample. A clear distinction in the tabular autoencoders is the difference in performance between treatment arms. The tabular model that obtained the best area is the denoising variant, with an area of 0.71±0.03 in the sunitinib arm. Meanwhile, the variational autoencoder achieved the worst score with 0.45±0.04 in the sunitinib arm. There was a significant difference when comparing the default autoencoder with the variational variant in the sunitinib arm (*p* = 9.01*e*^−9^).

A similar observation can be made for the graph autoencoders. The combination of sparse and denoising obtained the best score of 0.68 ± 0.06 in the TKI, whereas the variational autoencoder performed the worst with 0.48 ± 0.07 with the immunotherapy. Similarly, the variational autoencoder was significantly different from the default one (*p* = 1.6*e*^−4^). There is no significant difference between the tabular and graph autoencoders when no penalty is considered, meaning both models assign similar risks. However, there are differences between the avelumab + axitinib and sunitinib arms for all penalties except the variational one for both tabular and graph autoencoders.

Finally, Fig 6c shows the loss in PFS predictions among autoencoders. A similar observation to the previous figure can be made: overall, patients on sunitinib are predicted more accurately than those on avelumab + axitinib. The best variant within the tabular autoencoders is the sparse one, with a loss of 4.30 ± 0.95 in the sunitinib arm. Meanwhile, the variational autoencoder performs the worst, with a loss of 9.49 ± 0.85 in the avelumab + axitinib arm. Regarding graph autoencoders, they perform slightly better when working with the immunotherapy than tabular autoencoders do. The penalty that worked best on these autoencoders is the sparse penalty, with a loss of 3.45 ± 0.78, while the penalty that performed the worst is the variational one, with a loss of 10.16 ± 1.48. There were significant differences between the tabular and graph autoencoders in the sparse variant with the sunitinib arm (*p* = 4.4*e*^−2^), indicating that the graph model performs better than the tabular one in the TKI. Furthermore, there are differences between treatment arms for all penalties in both the tabular and graph autoencoders. One notable aspect regarding the PFS prediction is that our models tend to overestimate the survival of the patients. Around 70% of all cases were overestimated in terms of survival. Note that despite this overestimation, the predictions remained close to the actual values. We observe that the PCA model performed approximately 36% worse than the Sparse Autoencoder. The PFS prediction and ROC metrics for the PCA model are presented in the Appendix S1 Fig.

**Fig 7:**
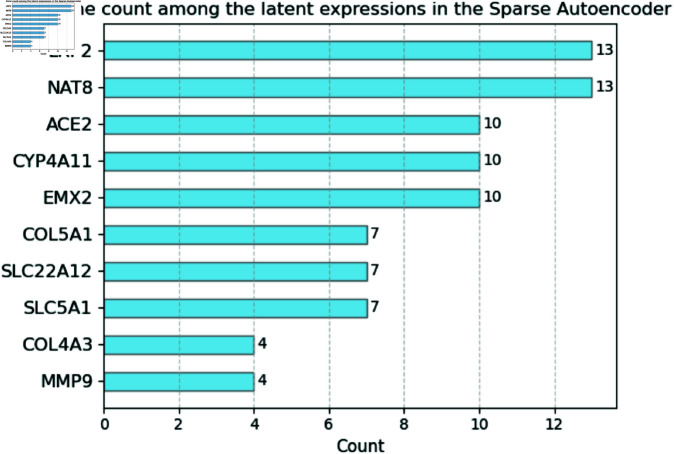
Frequency of each gene appearing in the top five latent features chosen by the statistical model over 10 folds for the sparse autoencoder.

Finally, we integrated interpretability into the models to identify which genes are most represented by the latent features. Considering the sparse autoencoder, which is among the models that obtained the best results and is known for finding meaningful representations, we obtained the five latent features with the highest coefficients assigned by the COX model. By finding the mutual information of each of these five latent features with the original data, we queried the top five genes most correlated to each representation. Fig 7 shows how many times each gene appeared in the most important latent features, with genes LRP2, NAT8, ACE2, CYP4A11, and EMX2 being the most frequent.

## Discussion

From the results presented, several key discussion points can be derived.

Firstly, despite the complexity of the data and the limited number of samples, autoencoders were able to faithfully reconstruct the original transcriptomic data. Modeling non-linear relationships also contributed to better predictions compared to using PCA. This confirms their suitability for compressing high-dimensional data of this nature. Notably, the tabular autoencoder consistently outperformed its graph counterpart in terms of reconstruction accuracy, despite both models sharing identical hyperparameters and similar architectures.

It is important to highlight that while the tabular autoencoder can model non-linear relationships between all genes, the graph autoencoder is constrained by the existing connections within the PPI network. Moreover, the accuracy of the PPI network is reliant on the specific genes selected via DisGeNet and the radius used to gather neighboring nodes, which makes the graph autoencoder’s performance highly dependent on the quality and structure of the PPI network, leading to variable reconstruction outcomes.

In terms of penalties, the denoising penalty yielded the best reconstruction results, particularly for the graph autoencoder. This is consistent with the nature of the denoising process, where the addition of noise helps the model generalize better [35]. On the other hand, the sparse autoencoder did not perform as well with graph data since its primary objective is to create meaningful representations by deactivating certain neural network weights [36]. However, combining sparse and denoising penalties resulted in improved reconstruction performance. Unfortunately, the variational autoencoder performed worse than the other methods in both reconstruction and prediction tasks. This can be attributed to the constraints imposed on the latent space to follow a specific distribution (in this case, Gaussian), which may result in information loss in the latent space [37]. Attempting to use a different distribution did not yield a better reconstruction either.

Beyond the machine learning models, valuable insights can also be drawn from the statistical model employed. The model is comprised of two components: the COX proportional hazards model, which estimates hazard ratios (and consequently risks), and Breslow’s estimator, a non-parametric method used to estimate survival functions. Although the PFS predictions were generally accurate, particularly for the graph autoencoder with a sparse penalty, a significant overestimation was observed. This overestimation stems from Breslow’s estimator, which is prone to inflate survival probabilities, especially when dealing with small sample sizes [38]. Increasing the sample size or selecting an alternative estimator could help alleviate this issue. Nevertheless, the semi-parametric model performed adequately, as demonstrated by the area under the ROC curve, though this was predominantly observed for the sunitinib arm. By contrast, the avelumab+axitinib arm’s performance closely resembled that of a random model.

In addition to making predictions, we identified the genes most prominently represented in the latent features produced by the sparse autoencoder, as illustrated in Fig 7. Among the five top genes associated with the prediction of PFS (LRP2, NAT8, ACE2, CYP4A11 and EMX2), it is interesting that all of them have been associated with RCC prognosis (PMIDs: 36851274,36980716, 34630525, 34976818, 34704468, 38183818, 37065178). Furthermore, LRP2, NAT8 and ACE2, have been shown to modulate the tumor microenvironment of RCC, a critical factor influencing the response of antiangiogenic drugs and immunotherapy. Specifically, LRP2 expression in RCC has been associated with a high tumor mutation burden and with the abundance of tumor-infiltrating immune cells (PMID 36851274). NAT8 expression has been shown to be regulated by methylation, and it has been connected with the infiltration of cancer-associated fibroblasts in kidney cancer (34630525). Regarding ACE2, this regulator of the renin-angiotensin system has been found to be expressed in the endothelial cells of the intratumor blood vessels (PMID: 28809959) and also connected with resistance to antiangiogenic drug treatment (PMID: 33296352). Thus, these genes seem to be critical regulators of RCC pathways connected with the aggressivity of the disease, but also with the tumor angiogenesis and immune cell infiltration.

A noteworthy discrepancy in performance was also observed between the avelumab+axitinib and sunitinib treatment arms, despite the fact that both arms had comparable transcriptomic profiles, PFS, and censoring data. One plausible explanation for this disparity is that the immunotherapy involves two distinct mechanisms (avelumab and axitinib), while sunitinib functions as a standalone agent. Predicting the response to a single mechanism is inherently less complex than predicting responses to a combination of therapies. A promising direction for future research could involve analyzing the differential response between sunitinib and each individual component of the immunotherapy regimen.

In summary, this study demonstrates the practicality of generating meaningful representations of complex, high-dimensional data in a reduced-dimensional space. Although further data compression is technically feasible, it would likely compromise the predictive performance of the statistical model.

It is important to acknowledge several limitations in our approach, which provide intriguing avenues for future work.

Firstly, increasing the amount of available data would not only improve the autoencoder’s reconstruction accuracy but also enhance the statistical model’s inference capabilities. Non-parametric and semi-parametric models are highly dependent on the observed data, making larger sample sizes critical for producing more reliable predictions.

Furthermore, the preprocessing steps, especially the creation of the PPI graphs, could be refined. In constructing these graphs, the genes selected from DisGeNet resulted in a network that was sparse and disconnected, necessitating the use of a neighbor search. However, some of these genes may not be directly relevant to the disease. A more selective and refined filtering process could ensure that only the most pertinent genes are included, thereby improving the quality of the PPI network.

As for the statistical model, while the COX elastic net proved to be an effective tool, future research could explore the use of parametric models. However, this would require a comprehensive understanding of the distribution of transcriptomic data to select an appropriate model.

In terms of interpretability, we sought to derive meaning from the latent representations based on the genes we analyzed. Other interpretability methods beyond mutual information, such as alternative techniques or visualizations of the gene latent space, could offer deeper insights into the data and enhance our understanding of the model’s decision-making process.

Overall, this work illustrates that autoencoders, when combined with statistical models, can yield valuable insights. While models such as the COX elastic net may take longer to produce results, the prior compression of the data proves advantageous, provided the model retains interpretability.

## Conclusion

We have conducted extensive research on different autoencoder types and variations to analyze their efficacy in predicting the response of two different treatment arms used to combat renal cell carcinoma. Despite the complexity of the data, autoencoders have proven to be excellent tools for compressing the dimensionality of the data into meaningful features, which can then be utilized by statistical approaches.

We believe that this methodology extends beyond renal cancer and holds promise for various medical fields, providing a robust approach to managing high-dimensional data and uncovering meaningful insights across different diseases. Although not yet suitable for direct clinical application, this tool has significant potential for patient screening, helping identify candidates for specific therapies. Its impact could be substantial in optimizing treatment processes, reducing costs, and ultimately improving patient survival.

This work offers multiple avenues for further expansion. Future research could validate our findings by analyzing a new patient cohort and refining the statistical approach by incorporating parametric estimators to enhance the model’s predictive accuracy.

## Supporting information

S1 Fig Autoencoder reconstruction results using an exponential distribution. PFS prediction and ROC metrics for the PCA model. (PNG)
