# Supplementary figures and images for: Autoencoder techniques for survival analysis on renal cell carcinoma
Source: PLoS One. 2025 May 15;20(5):e0321045. doi: 10.1371/journal.pone.0321045 (PMC12080797; doi:10.1371/journal.pone.0321045)

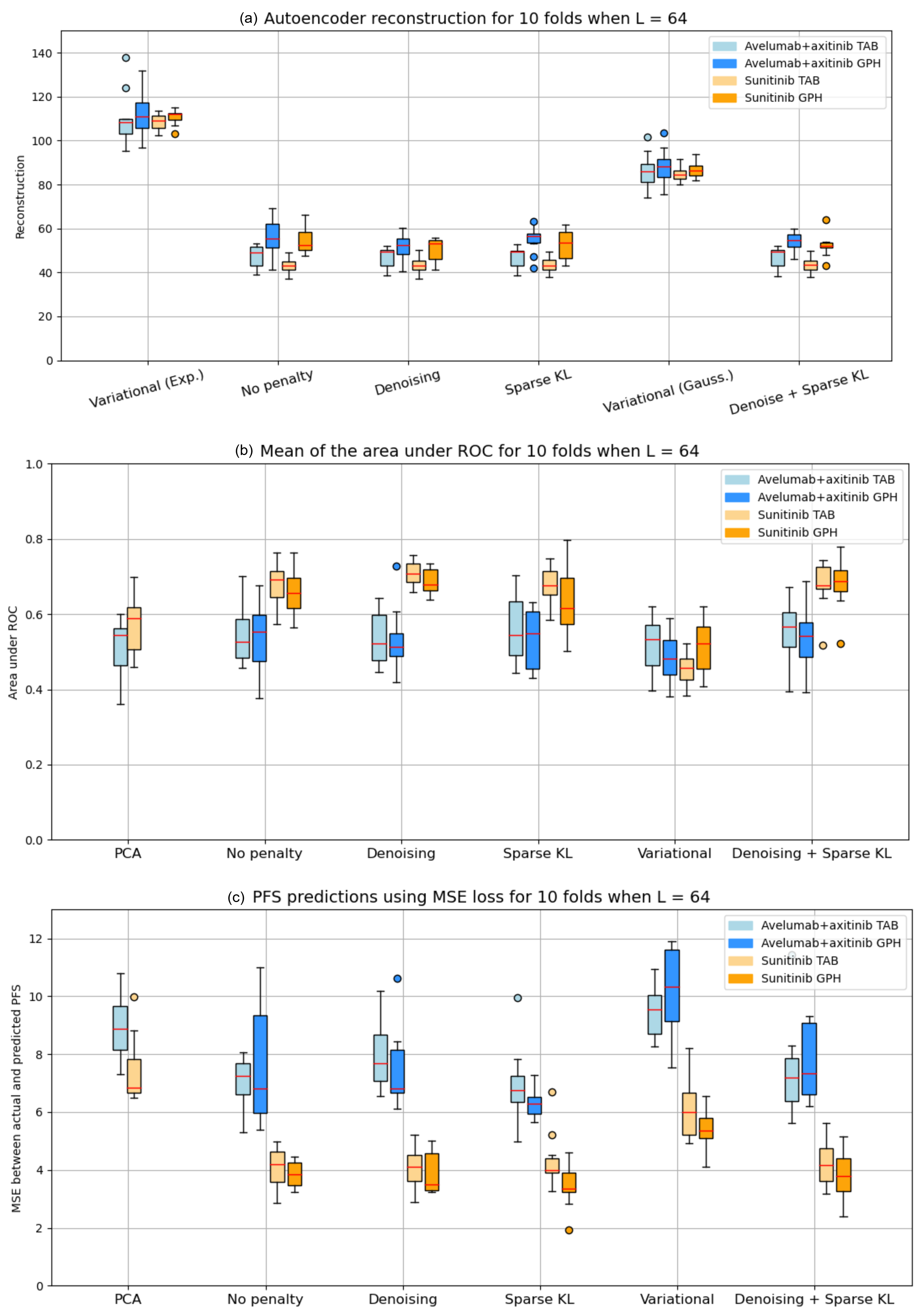

Supplement: S1 Fig — (PNG) [file pone.0321045.s001.png]
